# The Hardest Superconducting Metal Nitride

**DOI:** 10.1038/srep13733

**Published:** 2015-09-03

**Authors:** Shanmin Wang, Daniel Antonio, Xiaohui Yu, Jianzhong Zhang, Andrew L. Cornelius, Duanwei He, Yusheng Zhao

**Affiliations:** 1 HiPSEC & Physics Department, University of Nevada, Las Vegas, Nevada 89154, USA; 2 Institute of Atomic & Molecular Physics, Sichuan University, Chengdu 610065, China; 3 Los Alamos National Laboratory, Los Alamos, NM 87545, USA

## Abstract

Transition–metal (TM) nitrides are a class of compounds with a wide range of properties and applications. Hard superconducting nitrides are of particular interest for electronic applications under working conditions such as coating and high stress (e.g., electromechanical systems). However, most of the known TM nitrides crystallize in the rock–salt structure, a structure that is unfavorable to resist shear strain, and they exhibit relatively low indentation hardness, typically in the range of 10–20 GPa. Here, we report high–pressure synthesis of hexagonal δ–MoN and cubic γ–MoN through an ion–exchange reaction at 3.5 GPa. The final products are in the bulk form with crystallite sizes of 50 – 80 μm. Based on indentation testing on single crystals, hexagonal δ–MoN exhibits excellent hardness of ~30 GPa, which is 30% higher than cubic γ–MoN (~23 GPa) and is so far the hardest among the known metal nitrides. The hardness enhancement in hexagonal phase is attributed to extended covalently bonded Mo–N network than that in cubic phase. The measured superconducting transition temperatures for δ–MoN and cubic γ–MoN are 13.8 and 5.5 K, respectively, in good agreement with previous measurements.

Transition–metal (TM) mononitrides often crystallize in rock–salt structure and exhibit mixed metallic, ionic, and covalent bonding^1^,^2^. Depending on the exact nature of bonding, these nitrides possess a wide variety of properties including high hardness, high melting temperature, and high electronic conductivity^3^. However, despite the decades of experimental efforts, the intrinsic hardness has been limited to the level of 10–20 GPa^4^. This is not unexpected because, as commonly accepted, hardness is mainly attributed to the covalent bonding^5^,^6^, which is highly localized in substance and can effectively suppress the mobility of dislocations under indentation. The TM – N bonds in cubic nitrides are linearly distributed without involving three–dimensional (3D) network as formed in diamond and cubic–BN, the two well–known superhard materials. Such bonding characteristics are structurally unfavorable to resist severe shear strain or shape change, as manifested by their relatively low shear modulus of 130–200 GPa^4^,^7^.

The intrinsic hardness of a substance can be enhanced by tuning the covalent bonds. Filling of the covalent σ bonding states, for example, has been used for achieving high hardness in carbon–doped TM nitrides (*e.g.,* TiC_*x*_N_1-*x*_) with enhanced shear modulus^8^,^9^. Structural manipulation is another mechanism to alter materials properties. In addition to the common rock–salt structure, TM nitrides also crystallize in a number of different structures such as hexagonal MoN_*x*_^10^,^11^, orthorhombic Ta_3_N_5_^12^,^13^, rhombohedral W_2_N_3_^14^, and tetragonal Hf_3_N_4_^15^. Hexagonal δ–MoN is of particular interest because it adopts an unusual *P6*_*3*_*mc* structure (No. 186)^11^ with 3D cation–anion network, indicating ‘disordered’ atomic arrangements with highly directional bonds (ref. 16). δ–MoN is thus structurally more resistant against shear deformation than γ–MoN and, hence, has a potential to achieve higher hardness. Indeed, based on nano–indentation on thin–film coatings, δ–MoN exhibits a higher hardness of ~50 GPa than that of γ–MoN_*x*_ (~33 GPa). These measurements, however, were performed under an ultra–low load of 0.2 mN^17^, which would substantially overestimate the hardness values^18^. Although a value of ~25 GPa has recently been reported for δ–MoN using a Knoop indentation under an acceptable load of 0.2 N^19^, it is still challenging to accurately determine the intrinsic hardness of thin films of a few microns in thickness, owing to the influence of substrate and other extrinsic complications such as lattice strain and defects^18^,^20^.

δ–MoN exhibits superconducting transition at the T_c_ of 12–14 K^10^,^11^,^21^,^22^, which is the second highest among the known the metal nitrides and only slightly lower than the reported value of ~16 K for cubic γ–NbN^23^,^24^. The cubic γ–MoN is another long–sought candidate for high–temperature superconducting, and the predicted T_c_ is ~30 K^24^. However, measurements on traditional thin films show rather low T_c_ values of 4–6 K, and the discrepancy was attributed to the lattice disorder or substoichiometry in nitrogen^25^. To date, most reported synthesis products for δ– and γ–MoN are non–stoichiometric and poorly–crystallized in forms of thin films^17^,^19^,^22^, which severely limits their use in diverse scientific studies and technological applications.

A number of studies in the Zr–N^15^,^26^, Hf–N^26^, Ta–N^13^,^27^, and noble metal nitride systems^28^,^29^,^30^,^31^ have demonstrated the power of high–P synthesis in the search for new nitrides. Using a large volume press, we have recently synthesized stoichiometric CrN and a series of novel N–rich nitrides (*e.g.,* W_2_N_3_, W_3_N_4_, and MoN_2_)^14^,^32^,^33^,^34^, through newly formulated ion–exchange reactions at moderate pressures up to 5 GPa. In this work, we extended this high–P methodology to molybdenum mononitrides and have successfully synthesized stoichiometric δ– and γ–MoN with large crystallite sizes. With such improved specimen, we further determined their intrinsic hardness and superconducting transition temperature.

## Experimental Section

High–purity Na_2_MoO_4_ (>99.5% ~ 50 μm) and *h*BN (>99.9% ~ 50 μm) powders in the molar ratio Na_2_MoO_4_: BN = 1: 2 were homogeneously mixed and compacted into cylindrical pellets (12 mm in diameter and 10 mm in height) for the synthesis of molybdenum nitrides. In each experimental run, the pellet was contained in a molybdenum capsule to prevent potential contamination. High P–T synthesis was conducted using a DS 6 × 14 MN cubic press installed at Sichuan University, China^35^. For the synthesis of δ–MoN, the starting sample was quickly heated to 1300 °C within 3–5 min (~300 °C /min) at 3.5–5 GPa, and then soaked for 20 min before quenching to room temperature. To obtain metastable γ phase, the reactants were slowly heated to 1600 °C at a rate of ~5 °C/min, followed by quenching to room temperature. Such prolonged heating would facilitate nucleation of γ–MoN in the P–T region where it is thermodynamically favorable. The run products were washed with distilled water to remove the byproduct NaBO_2_ and unreacted Na_2_MoO_4_ (Suppl Figure S1), followed by drying in an oven at 348 K. To grow large single crystals for δ phase, the phase–pure δ–MoN powders were re–sintered at 5–8 GPa and 1400–1800 °C for 30–60 min, using a Kawai–type high–P apparatus installed at Arizona State University^36^.

The final run products were characterized by x–ray diffraction (XRD) with Cu *Kα* radiation, optical microscopy, field emission scanning electron microscopy (SEM), and energy–dispersive x–ray (EDX) analysis. High*–*pressure angle–dispersive synchrotron XRD experiments using a diamond–anvil cell (DAC) were performed up to 60 GPa at the HPCAT 16BM–D beamline of the Advance Photon Source (APS). The nitride powders were loaded into the sample hole in a rhenium gasket pre–indented to ~30 micron thickness with helium as the pressure–transmitting medium. A few ruby balls were also loaded in the sample chamber to serve as the internal pressure standard. The experimental details have been described previously^14^.

Low–temperature a.c. magnetic susceptibility and four–probe resistivity measurements were performed on powdered and sintered bulk samples, respectively, using a commercially available Quantum Design PPMS. For magnetic susceptibility measurement, the powdered sample was sealed in a plastic capsule. An oscillating 1000 Hz magnetic field peak strength of 10 Oe was applied to the sample as it was field–cooled down to 2 K in a static background field up to 3 Tesla.

Vickers hardness was measured on single–crystal δ– and γ–MoN under different loads of 25, 50, 100, and 200 g by using a Micromet–2103 hardness tester (Buehler, USA). Under each applied load, the measurement was performed with a dwelling time of 15 s, and was repeated 5–10 times to obtain statistically improved averages. Before measurements, the specimen was mounted on a SiO_2_ or an Al_2_O_3_ substrate using the epoxy resin, and the mirror–quality surfaces were prepared for the measurement.

## Results and Discussion

**Figure 1:**
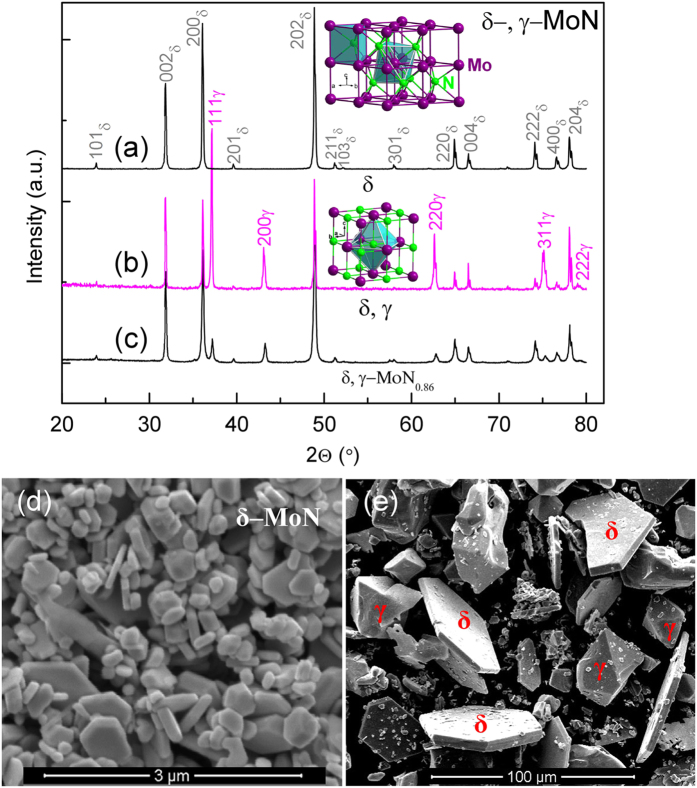
(**a**–**c**) XRD patterns collected at ambient conditions with a copper radiation target. SEM images corresponding to (**a**,**b**) are shown in (**d**,**e**). The run product in (**a**,**d**) is phase–pure hexagonal δ–MoN synthesized at ~5 GPa and ~1300 °C for 20 min. (**b**,**e**) show mixed γ– and δ–MoN phases synthesized at 3.5 GPa by program–controlled heating for 3 hours (see Experimental Section). (**c**) Cubic γ–MoN_0.86_ obtained from re–sintering of phase–pure δ–MoN in (**a**,**d**) at ~5 GPa and ~2200 °C for 15 s. Insets show polyhedral views of crystal structures for δ and γ phases.

Figure 1a shows an x–ray diffraction (XRD) pattern of the run product purified from the synthesis at 5 GPa and 1300 °C for 20 min. All Bragg peaks in Fig. 1a can be indexed by a hexagonal unit cell with space group of *P6*_*3*_*mc* (No. 186) for δ–MoN. The refined lattice parameters are *a* = 5.7417 (5) Å and *c* = 5.6187 (3) Å, close to previously reported values of *a* = 5.7366 Å and *c* = 5.6188 Å^11^. For metastable γ–MoN, successful synthesis has only been made in the form of thin films at relatively low temperatures^17^,^19^,^37^. To obtain this phase in the bulk form, we slowly heated the sample cell up to 1600 °C at 3.5 GPa (see Experimental Section). As shown in Fig. 1b, the cubic γ–MoN forms as a major phase and coexists with δ–MoN. The refined lattice parameter for γ–MoN is *a* = 4.1925 (3) Å, close to reported values for stoichiometric thin films (~4.21 Å)^38^,^39^. The slight expansion in the lattice parameter of thin films may be attributed to atomic disorder and/or substrate materials^38^,^39^.

Both δ– and γ–MoN were formed through the following solid–state reaction,


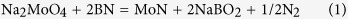


which can simply be viewed as ion exchange between Mo^6+^ and B^3+^^14^,^32^,^40^, ^41^,^42^. As expected, compositional analyses using EDX show the same Mo: N molar ratio for both phases. Combined with the lattice–parameter refinements discussed in the preceding paragraph, we conclude that the high–P synthesized δ– and γ–MoN are stoichiometric. Compared with γ–MoN, the δ phase adopts unusual anion–cation coordination: the Mo atoms are octahedrally coordinated with N atoms, [NMo_6_], and the N atoms are trigonal–prismatically coordinated with Mo atoms, [MoN_6_] (see insets in Fig. 1 and Suppl Figure S4). In addition, a sub–stoichiometric γ–MoN_0.86_ phase (in Fig. 1c) was produced by subjecting phase–pure δ–MoN to high–T treatment at ~5 GPa and ~2200 °C for 15 seconds. The nitrogen concentration *x* in γ–MoN_*x*_ can be estimated from the *a*–*x* relationship (Suppl Figures S2–S3).

Figure 1d shows a typical scanning electron microscopy (SEM) image of purified δ–MoN (see XRD pattern in Fig. 1a). The sample is well–crystallized with crystallite size of 0.2–1 μm. The layered and plate–like morphology originates from its hexagonal structure. For the sample synthesized using a prolonged heating procedure, as shown in Fig. 1e, two different crystal forms of hexagons and octahedrons correspond to δ– and γ–MoN (also see Fig. 1b and Suppl Figure S5), respectively, indicating that the crystal growth is along the (002) plane in δ–MoN and (111) plane in γ–MoN. Strikingly, γ–MoN exhibits large crystal sizes of 30–80 μm, suitable for Vickers hardness measurement on a single crystal. For δ phase, the crystal thickness is only 4–5 μm, which as mentioned above, is technically inadequate for accurate measurement of the hardness. To grow larger crystals, the phase–pure δ–MoN powders were re–sintered at 5–8 GPa and 1400–1800 °C. Different from the starting morphology, crystals in the re–sintered sample show more uniform geometries suitable for hardness measurement (see Suppl Figure 5).

**Figure 2:**
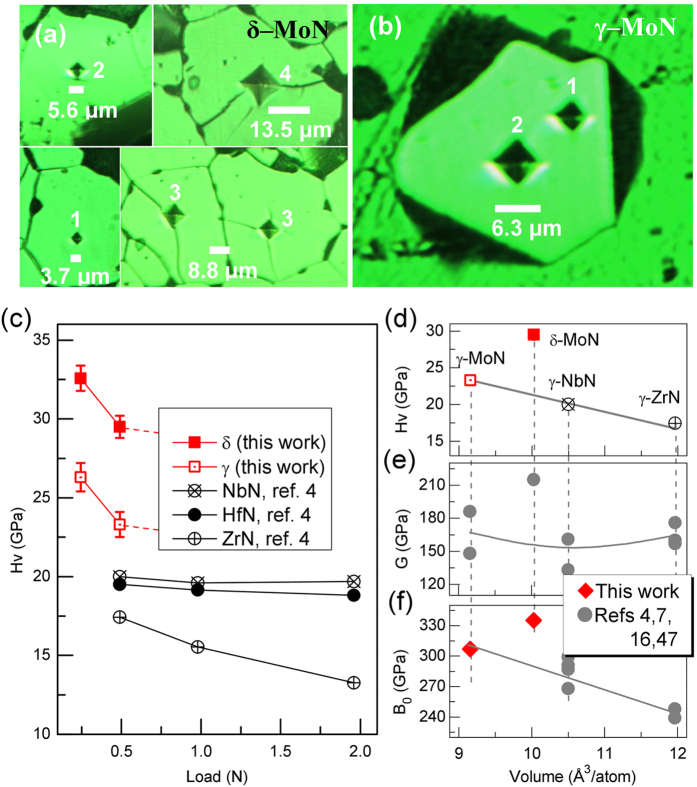
(**a**,**b)** Vickers hardness measurement for single–crystal δ– and γ–MoN. Indentations numbered with ′1′, ′2′, ′3′, and ′4′ correspond to applied loads of 0.245, 0.49, 0.98, and 1.96 N, respectively. The crystals in (**a**) were prepared by sintering of phase–pure δ–MoN powders at 8 GPa and 1800 °C for 60 min. The γ–MoN crystal in (**b**) was directly grown using ion–exchange reaction (see Fig. 1e). (**c**) Vickers hardness, Hv, of δ– and γ–MoN as a function of applied load. Also plotted are the reported Hv values for other hard nitrides including ZrN, NbN, and HfN (ref. 4). (**d**) Hv *vs* volume per atom (*i.e.,* normalized volume in terms of per atom) for γ–MoN, γ–ZrN (ref. 4), and γ–NbN (ref. 4) under a load of 0.49 N. (**e**) Bulk modulus, B_0_, *vs* volume per atom. The measured bulk moduli for δ– and γ–MoN are ~335 and ~307 GPa, respectively, based on compression experiments (Suppl Figures S6–S7). (**f**) Shear modulus, G, *vs* volume per atom.

Figure 2a,b show typical indentation pyramids on the δ– and γ–MoN single crystals with randomly oriented surfaces. The measured Vickers hardness, Hv, is plotted in Fig. 2c as a function of load. Using asymptotic leveling as a criterion, the Hv values can appropriately be determined for both phases under the loads of 0.245 and 0.49 N. Also plotted in Fig. 2c are a number of recently reported values for cubic nitrides including ZrN, NbN, and HfN, all of which were obtained from micro–indentation (*i.e.,* Vickers hardness) on single–crystal samples^4^. Compared with the known superhard materials such as cubic BN and BC_2_N (ref. 43), the Hv values in all these nitrides are leveled off at a much smaller critical load of 0.49 N, indicating that the plastic deformation starts to prevail (ref. 18). The thus–determined asymptotic Hv values for both δ– and γ–MoN are ~30 GPa and ~23 GPa, respectively, which are so far the two hardest metal nitrides. In particular, the hardness of δ–MoN surpasses that of moissanite (SiC ~ 26 GPa)^44^ and WC (~22 GPa)^45^, and approaches that of WB_4_^46^, one of the hardest TM borides. Even in the case that entire crystal was severely cracked at loads exceeding 0.49 N (see Fig. 2a), the obtained Hv values for δ–MoN are still in the range of 20–25 GPa.

To understand the hardness enhancement in MoN, the Hv, bulk modulus (B_0_), and shear modulus (G) of δ–MoN and isostructural γ–MoN, γ–NbN, and γ–ZrN are plotted as a function of ambient volume per atom (V_0_), as shown in Fig. 2d,e,f. δ–MoN is a low–density phase and is ~9.5% less dense than the γ phase. For cubic γ nitrides, the asymptotic Hv values increase linearly with decreasing V_0_ (Fig. 2d). In contrast, δ–MoN deviates substantially from this trend and stands out to be the hardest metal nitride. The enhanced hardness in δ–MoN is mainly attributed to its 3D anion–cation bonding network, which has a greater ability to withstand shear deformation. Indeed, the calculated shear modulus for δ–MoN (~220 GPa) is ~35% greater than those of γ nitrides (~160 GPa) with linearly distributed bonds^4^,^7^,^16^,^47^. In addition, compared with cubic phase, the more strongly covalent and directional bonding in δ–MoN has recently been explored^16^, using the first–principles calculations.

Figure 2f shows the bulk modulus data of these nitrides. The low–density δ–MoN possesses a high *B*_*0*_ of ~335 GPa and is more than 9% larger than that of high – density γ–MoN (~307 GPa) (Suppl Figure S7). This anomalous elastic strengthening in the low–density phase has only sparsely been reported in other material systems^48^,^49^, which is presumably attributed to the extended 3D covalent bonding network in δ–MoN. In addition, the stiffness of *c*–axis in δ–MoN exceeds that in WC, one of the most incompressible substances, and is nearly twice that of cubic γ–MoN (see Suppl Figure S8 and Table S1). For γ nitrides, the *B*_*0*_ satisfies the general relation, *B*_*0*_ ∝ 1/V_0_, as commonly observed in other isostructural systems^18^,^50^. As shown in Fig. 1d,f, although *B*_*0*_ and Hv show similar trends of variation with the volume for cubic nitrides, such correlations should not be overstated because hardness is ultimately a measure of plastic deformation and only weakly correlated with the elastic bulk modulus. On the other hand, the bonding in γ–MoN should be significantly enhanced due to the shortened cation–anion distance, which eventually leads to a high hardness in γ–MoN when compared with other cubic metal nitrides (see Fig. 2d). It is worthwhile to mention that the measured B_0_ for sub–stoichiometric γ–MoN_0.86_ is ~302 GPa, and close to those of γ–MoN (~307 GPa) (Suppl Figure S7) and γ–MoN_0.5_ (~301 GPa in ref. 51), indicating that in the range of *x* = 0.5–1 the elastic modulus of γ–MoN_*x*_ is insensitive to the nitrogen concentration. The similar elastic behavior has previously been reported in other transition–metal carbide and nitride systems (such as NbC_*x*_)^9^ and has been explained in terms of chemical bond^52^. Because of nitrogen vacancies in those substoichiometric compounds, the anion–cation covalent *p–d* bond is softened. Compared with metallic d–d bond, the fraction of such *p–d* bond is also increased due to substoichiometry^52^, leading to a slow variation of elastic modulus with nitrogen concentration.

**Figure 3:**
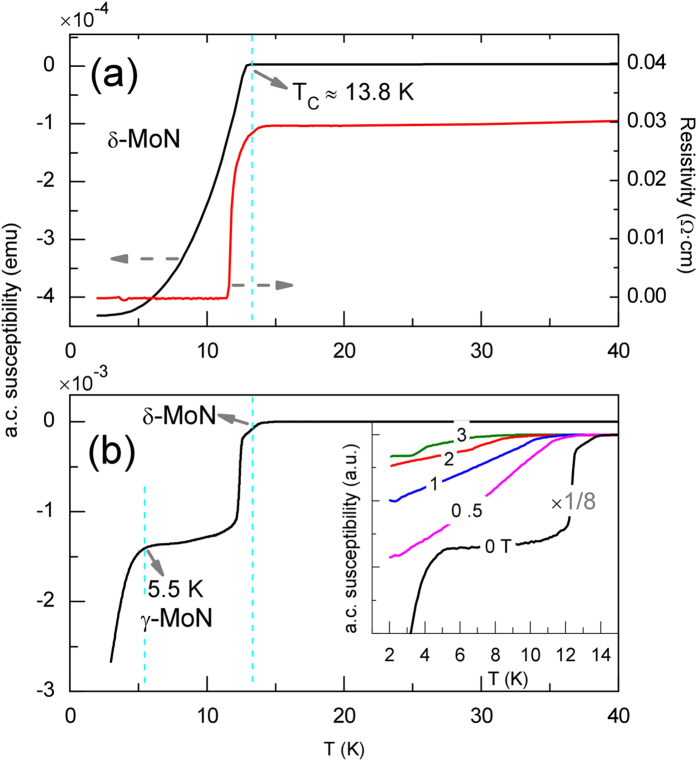
Low–temperature magnetic susceptibility measurement on (a) phase–pure δ–MoN and (b) a mixture of δ and γ phases (see Fig. 1b,e). Inset in (**b**) shows the data collected under different magnetic fields of H = 0, 0.5, 1, 2, and 3 T. Also plotted in (**a**) are electrical resistivity measurements for the sintered δ–MoN polycrystalline bulk sample (see Fig. 2a).

From low–T a. c. susceptibility and four–probe resistivity measurements, the determined T_C_ and transition width, ΔT_c_, for the well–crystallized δ–MoN are ~13.8 K and 2.3 K (see Fig. 3a and Suppl Figure S9), respectively, both of which are higher than those reported for thin films (~13 K and 0.3 K)^22^, mainly due to the non–stoichiometry and atomic disorder in thin films as superconductivity is sensitive to crystal defects. Above the T_c_, δ–MoN is metallic and shows weak electrical resistance (~0.03–0.05 Ω cm) with a positive temperature coefficient of resistivity (TCR) (Suppl Figure S9). For sample with coexisting δ– and γ–MoN (see Fig. 1e), the magnetic susceptibility shows substantial drops near ~13.8 K and 5.5 K as temperature decreases, corresponding to the superconducting transitions in δ– and γ–MoN, respectively. Despite the fact that γ–MoN is stoichiometric and well–crystallized, the measured T_c_ (5.5 K) is still rather low, and it agrees well with previously reported values (4–6 K) for the γ–MoN_*x*_ thin films with *x* ≈ 1^38^,^39^. Clearly, all experimentally determined T_c_ values are considerably lower than the predicted T_c_ of ~30 K. This discrepancy is likely due to magnetic instabilities of γ–MoN that may destroy superconductivity^24^,^25^. Besides, the T_c_ of cubic phase is more sensitive to applied field than that of hexagonal phase (see Suppl Figure S10 for detail). In fact, search for new materials with excellent hardness and superconductivity is highly demanded from the perspectives of such technological applications as superconducting nano–electromechanical systems and high–pressure devices^53^.

TM mononitrides hold great promise for achieving the highest hardness and high–T_c_ superconductivity in the nitride systems. Deviation from the 1:1 metal/nitrogen ratio would enhance metallic bonding (e.g., Mo_2_N) or lead to layered structures (e.g., 3 R–MoN_2_)^34^ involving the weak van der Waals bonding, either of which will have negative impact on hardness and superconductivity. Recent high–P synthesis efforts have discovered several novel TM nitrides or their polymorphs, including orthorhombic Ta_2_N_3_^27^ and Ta_3_N_5_^13^, cubic A_3_N_4_ (A = Zr and Hf)^27^, and tetragonal and orthorhombic Hf_3_N_4_^15^. However, the metal cations in these nitrides are 7–9 fold coordinated with nitrogen anions (*i.e.*, *x* = 7–9 in [TMN_*x*_]), which would lead to spatially uniform distribution of electron density and is hence unfavorable for the formation of directional bonding to withstand shear deformation under indentation^18^. From this viewpoint, the zincblende–type nitrides, XN (X = Fe, Co, and Pt)^31^,^54^,^55^, may possess excellent hardness because metal ions are tetragonally coordinated with N, [TMN_4_], which forms directional bonds, resembling those of diamond and cubic–BN. Further experimental efforts along this direction are appealing for exploring novel metal nitrides with superior hardness and high–T_C_ superconductivity.

In summary, stoichiometric hexagonal δ– and cubic γ–MoN were synthesized through an ion–exchange reaction route at high pressures. Based on single crystal measurements, δ– and γ–MoN exhibit high asymptotic hardness of ~30 and 23 GPa, respectively. Consistent with previous studies, the measured superconducting transition temperatures of 13.8 K for δ–MoN and 5.5 K for γ–MoN. δ–MoN is so far the hardest metal nitride with the second highest T_c_, comparable to that of NbN (~16 K). The enhanced hardness in δ phase is attributed to three–dimensional, covalent Mo–N bonding network. In contrast, the Mo–N bonds in γ–MoN are linearly distributed and structurally unfavorable to achieve high hardness. Although δ–MoN is a low–density phase, it exhibits an anomalously higher elastic modulus than the high–density γ phase. Phase–pure δ–MoN can readily be synthesized at a moderate pressure of 3.5 GPa, making it practically feasible for massive and industrial–scale production.

## Additional Information

**How to cite this article**: Wang, S. *et al.* The Hardest Superconducting Metal Nitride. *Sci. Rep.* **5**, 13733; doi: 10.1038/srep13733 (2015).

## Supplementary Material

Supplementary Information

## References

[b1] Papaconstantopoulos D. A., Pickett W. E., Klein B. M. & Boyer L. L. Electronic properties of transition-metal nitrides: The group-V and group-VI nitrides VN, NbN, TaN, CrN, MoN, and WN. Phys. Rev. B 31, 752–761 (1985). 10.1103/physrevb.31.752 9935816

[b2] Blaha P., Redinger J. & Schwarz K. Bonding study of TiC and TiN. II. Theory. Phys. Rev. B 31, 2316–2325 (1985). 10.1103/physrevb.31.2316 9936039

[b3] Pierson H. O. in Handbook of Refractory Carbides and Nitrides (William Andrew Publishing, 1996).

[b4] Chen X.-J. *et al.* Hard superconducting nitrides. Proc. Nat. Acad. Sci. USA 102, 3198–3201 (2005). 15728352 10.1073/pnas.0500174102 PMC552926

[b5] Gilman J. J. Physical chemistry of intrinsic hardness. Mater. Sci. Eng. A 209, 74–81 (1996).

[b6] Kaner R. B., Gilman J. J. & Tolbert S. H. Designing superhard materials. Science 308, 1268–1269 (2005). 15919983 10.1126/science.1109830

[b7] Fulcher B. D., Cui X. Y., Delley B. & Stampfl C. Hardness analysis of cubic metal mononitrides from first principles. Phys. Rev. B 85, 184106 (2012).

[b8] Jhi S.-H., Ihm J., Louie S. G. & Cohen M. L. Electronic mechanism of hardness enhancement in transition-metal carbonitrides. Nature 399, 132–134 (1999).

[b9] Holleck H. Material selection for hard coatings. J. Vac. Sci. Technol. A 4, 2661–2669 (1986).

[b10] Ganin A. Y., Kienle L. & Vajenine G. V. Synthesis and characterisation of hexagonal molybdenum nitrides. J. Solid State Chem. 179, 2339–2348 (2006).

[b11] Bull C. L., McMillan P. F., Soignard E. & Leinenweber K. Determination of the crystal structure of δ-MoN by neutron diffraction. J. Solid State Chem. 177, 1488–1492 (2004).

[b12] Takata T., Lu D. & Domen K. Synthesis of structurally defined Ta_3_N_5_ particles by flux-assisted nitridation. Cryst. Growth Des. 11, 33–38 (2011).

[b13] Salamat A. *et al.* Synthesis of U_3_Se_5_ and U_3_Te_5_ type polymorphs of Ta_3_N_5_ by combining high pressure-temperature pathways with a chemical precursor approach. Chem. Commun. 50, 10041–10044 (2014). 10.1039/c4cc05147e 25051155

[b14] Wang S. *et al.* Synthesis, crystal structure, and elastic properties of novel tungsten nitrides. Chem. Mater. 24, 3023–3028 (2012).

[b15] Salamat A. *et al.* Synthesis of tetragonal and orthorhombic polymorphs of Hf_3_N_4_ by high-pressure annealing of a prestructured nanocrystalline precursor. J. Am. Chem. Soc. 135, 9503–9511 (2013). 23721167 10.1021/ja403368b PMC3715886

[b16] Kanoun M. B., Goumri-Said S. & Jaouen M. Structure and mechanical stability of molybdenum nitrides: A first-principles study. Phys. Rev. B 76, 134109 (2007).

[b17] Kazmanli M. K., Ürgen M. & Cakir A. F. Effect of nitrogen pressure, bias voltage and substrate temperature on the phase structure of Mo–N coatings produced by cathodic arc PVD. Surf. Coat. Technol. 167, 77–82 (2003).

[b18] Brazhkin V. V., Lyapin A. G. & Hemley R. J. Harder than diamond: Dreams and reality. Philos. Mag. A 82, 231–253 (2002).

[b19] Zhu X. *et al.* Phase composition and tribological performance of molybdenum nitride coatings synthesized by IBAD. Surf. Coat. Technol. 228, Supplement 1, S184–S189 (2013).

[b20] Ma Z. S., Zhou Y. C., Long S. G. & Lu C. On the intrinsic hardness of a metallic film/substrate system: Indentation size and substrate effects. Int. J. Plast. 34, 1–11 (2012).

[b21] Inumaru K., Baba K. & Yamanaka S. Structural distortion and suppression of superconductivity in stoichiometric B1-MoN epitaxial thin films. Phys. Rev. B 73, 052504 (2006).

[b22] Zhang Y. *et al.* Epitaxial superconducting δ-MoN films grown by a chemical solution method. J. Am. Chem. Soc. 133, 20735–20737 (2011). 22126391 10.1021/ja208868k

[b23] Bailey E. & McMillan P. F. High pressure synthesis of superconducting nitrides in the MoN-NbN system. J. Mater. Chem. 20, 4176–4182 (2010).

[b24] Papaconstantopoulos D. A., Pickett W. E., Klein B. M. & Boyer L. L. Superconductivity: Nitride offers 30K transition? Nature 308, 494–495 (1984).

[b25] Hart G. L. W. & Klein B. M. Phonon and elastic instabilities in MoC and MoN. Phys. Rev. B 61, 3151–3154 (2000).

[b26] Zerr A., Miehe G. & Riedel R. Synthesis of cubic zirconium and hafnium nitride having Th_3_P_4_ structure. Nature Mater. 2, 185–189 (2003). 12612677 10.1038/nmat836

[b27] Zerr A. *et al.* High-pressure synthesis of tantalum nitride having orthorhombic U_2_S_3_-type structure. Adv. Funct. Mater. 19, 2282–2288 (2009).

[b28] Friedrich A. *et al.* Novel rhenium nitrides. Phys. Rev. Lett. 105, 085504 (2010). 20868112 10.1103/PhysRevLett.105.085504

[b29] Young A. F. *et al.* Synthesis of novel transition metal nitrides IrN_2_ and OsN_2_. Phys. Rev. Lett. 96, 155501 (2006). 16712167 10.1103/PhysRevLett.96.155501

[b30] Crowhurst J. C. *et al.* Synthesis and characterization of the nitrides of platinum and iridium. Science 311, 1275–1278 (2006). 16513980 10.1126/science.1121813

[b31] Gregoryanz E. *et al.* Synthesis and characterization of a binary noble metal nitride. Nature Mater. 3, 294–297 (2004). 15107839 10.1038/nmat1115

[b32] Chen M. *et al.* Synthesis of stoichiometric and bulk CrN through a solid-state ion-exchange reaction. Chem. Eur. J. 18, 15459–15463 (2012). 23059561 10.1002/chem.201202197

[b33] Wang S. *et al.* Experimental invalidation of phase-transition-induced elastic softening in CrN. Phys. Rev. B 86, 064111 (2012).

[b34] Wang S. *et al.* A new molybdenum nitride catalyst with rhombohedral MoS_2_ structure for hydrogenation applications. J. Am. Chem. Soc. 137, 4815–4822 (2015). 25799018 10.1021/jacs.5b01446

[b35] Wang S., He D., Wang W. & Lei L. Pressure calibration for the cubic press by differential thermal analysis and the high-pressure fusion curve of aluminum. High Pressure Res. 29, 806–814 (2009).

[b36] Leinenweber K. D. *et al.* Cell assemblies for reproducible multi-anvil experiments (the COMPRES assemblies). Am. Mineral. 97, 353–368 (2012).

[b37] Lai G. C., Takahashi M., Nobugai K. & Kanamaru F. Phase transition in B1-type Mo_1−x_Nb_x_N sputtered films under ammonia annealing. J. Solid State Chem. 82, 1–7 (1989).

[b38] Linker G., Smithey R. & Meyer O. Superconductivity in MoN films with NaCl structure. J. Phys. F 14, L115–L119 (1984).

[b39] Savvides N. High Tc superconducting B1 phase MoN films prepared by low-energy ion-assisted deposition. J. Appl. Phys. 62, 600–610 (1987).

[b40] Lei L. & He D. Synthesis of GaN crystals through solid-state metathesis reaction under high pressure. Cryst. Growth Des. 9, 1264–1266 (2009).

[b41] Lei L. *et al.* Synthetic route to metal nitrides: high-pressure solid-state metathesis reaction. Inorg. Chem. 52, 13356–13362 (2013). 24251987 10.1021/ic4014834

[b42] Ma H. *et al.* GaN crystals prepared through solid-state metathesis reaction from NaGaO_2_ and BN under high pressure and high temperature. *J. Alloy. Co*mpd. 509, L124–L127 (2011).

[b43] Zhao Y. *et al.* Superhard B–C–N materials synthesized in nanostructured bulks. J. Mater. Res. 17, 3139–3145 (2002).

[b44] Qian J., Daemen L. L. & Zhao Y. Hardness and fracture toughness of moissanite. Diamond Relat. Mater. 14, 1669–1672 (2005).

[b45] Takahashi T. & Freise E. J. Determination of the slip systems in single crystals of tungsten monocarbide. Philos. Mag. 12, 1–8 (1965).

[b46] Mohammadi R. *et al.* Tungsten tetraboride, an inexpensive superhard material. Proc. Nat. Acad. Sci. USA. 108, 10958–10962 (2011). 21690363 10.1073/pnas.1102636108 PMC3131357

[b47] Yang Z.-X. *et al.* The mechanical properties of MoN under high pressure and effect of metallic bonding on its hardness. Solid State Sci. 28, 20–25 (2014).

[b48] Wang S. *et al.* Phase-transition induced elastic softening and band gap transition in semiconducting PbS at high pressure. Inorg. Chem. 52, 8638–8643 (2013). 23909959 10.1021/ic400801s

[b49] Wang S. *et al.* Revisit of pressure-induced phase transition in PbSe: crystal structure, and thermoelastic and electrical properties. Inorg. Chem. 54, 4981–4989 (2015). 25938257 10.1021/acs.inorgchem.5b00591

[b50] Zhang J. & Reeder R. J. Comparative compressibilities of calcite-structure carbonates; deviations from empirical relations. Am. Mineral. 84, 861–870 (1999).

[b51] Soignard E. *et al.* High-pressure synthesis and study of low-compressibility molybdenum nitride (MoN and MoN_(1−x)_) phases. Phys. Rev. B 68, 132101 (2003).

[b52] Jhi S.-H., Louie S. G., Cohen M. L. & Ihm J. Vacancy hardening and softening in transition metal carbides and nitrides. Phys. Rev. Lett. 86, 3348–3351 (2001). 11327967 10.1103/PhysRevLett.86.3348

[b53] Blase X. *et al.* Superconducting group-IV semiconductors. Nature Mater. 8, 375–382 (2009). 19387452 10.1038/nmat2425

[b54] Suzuki K. *et al.* Neutron diffraction studies of the compounds MnN and FeN. J. Phys. Soc. Jpn. 70, 1084–1089 (2001).

[b55] Suzuki K. *et al.* Crystal structure and magnetic properties of the compound CoN. J. Alloys Compd. 224, 232–236 (1995).

