# Supplementary material for: The Hardest Superconducting Metal Nitride
Source: Sci Rep. 2015 Sep 3;5:13733. doi: 10.1038/srep13733 (PMC4558542; doi:10.1038/srep13733)
Supplement: Supplementary Information [file srep13733-s1.pdf]

Supplementary Information

The Hardest Superconducting Metal Nitride

Shanmin Wang,[1,2,3,] Daniel Antonio,[1] Xiaohui Yu,[3] Jianzhong Zhang,[3] Andrew L. Cornelius,[1] Duanwei He,[2] and Yusheng Zhao[1,3,*]*

[1]*HiPSEC & Physics Department, University of Nevada, Las Vegas, Nevada 89154, USA*
[2]*Institute of Atomic & Molecular Physics, Sichuan University, Chengdu 610065, China*
[3]*Los Alamos National Laboratory, Los Alamos, NM 87545, USA*

[*] *E-mails: ShanminWang@gmail.com (S. Wang) and Yusheng.Zhao@UNLV.edu (Y. Zhao)

1. Synthesis, crystal structure, and stoichiometry

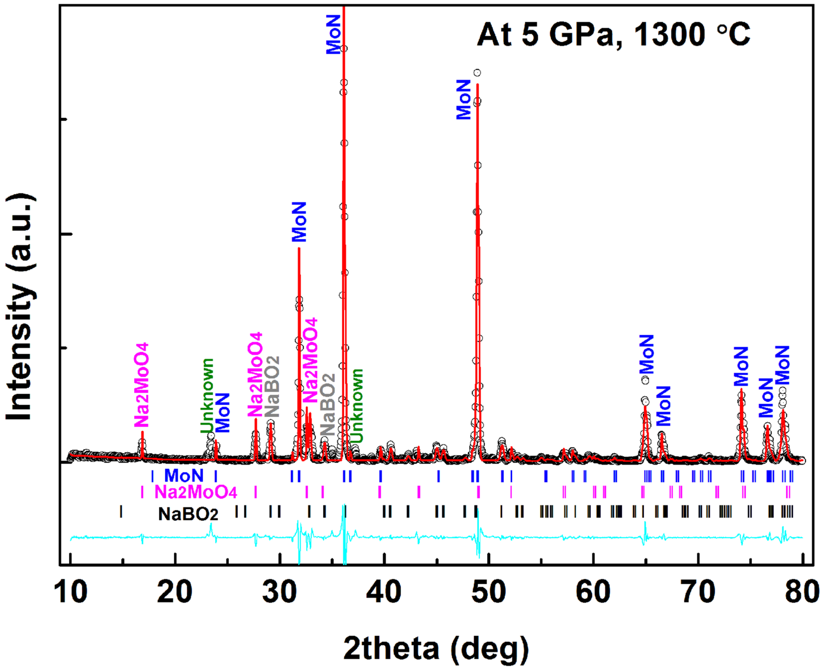

Fig. S1. An XRD pattern of the experimental run product synthesized at 5 GPa and 1300 °C for 20 min. The refinement was performed using Le Bail analysis. Apart from δ–MoN, the byproduct $NaBO_2$ and unreacted Na_2MoO_4 are identified, both of which can readily be removed by washing the product in distilled water.

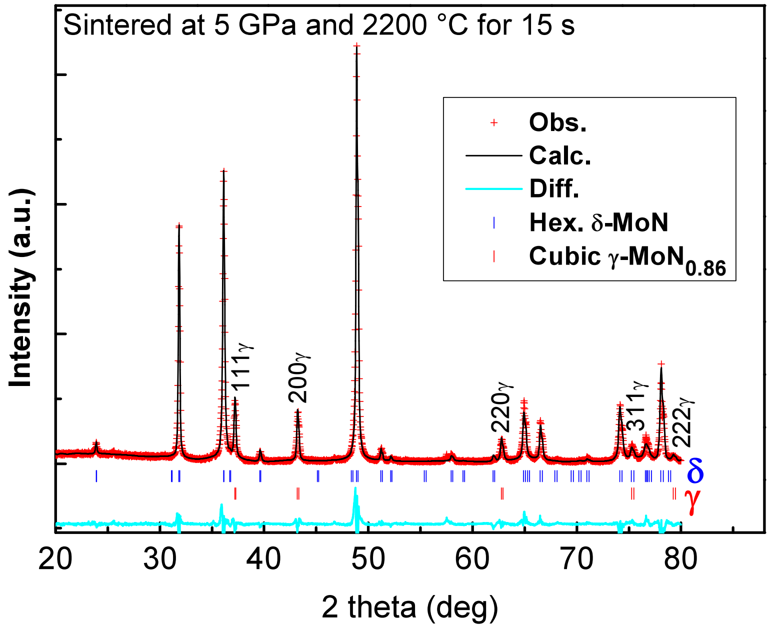

Fig. S2. The refined XRD pattern for the run product with co–existing δ–MoN and γ–$MoN_{0.86}$ phases. The data were collected at ambient conditions with a copper target. The sample was prepared by sintering phase–pure δ–MoN at ~5 GPa and ~2200 °C for 15 s. The crystal structures for both phases are refined using the GSAS software, based on the Rietveld method.[1]

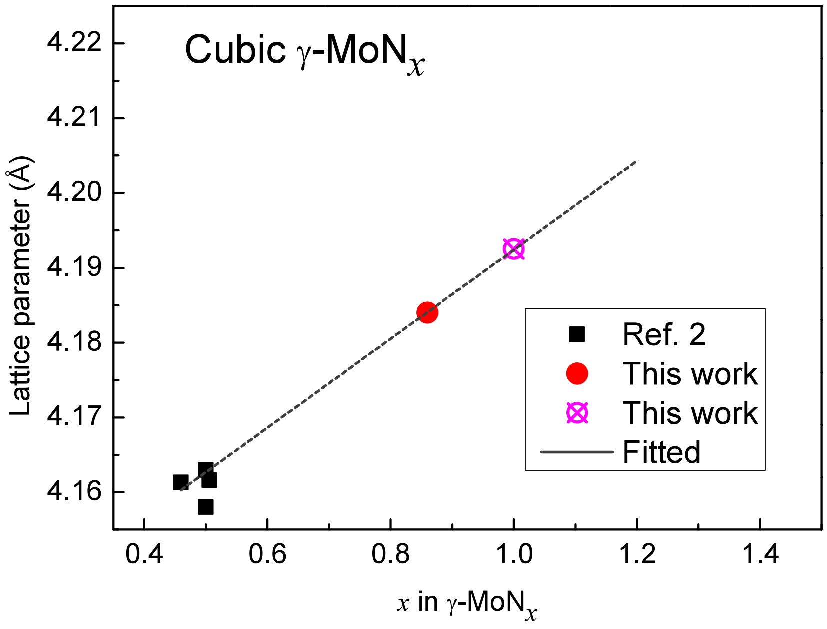

Fig. S3. Lattice parameter, *a*, as a function of *x* in γ–MoN_x. The black dots represent reported lattice parameter for substoichiometric γ–MoN_x (x = 0.46 – 0.51) [2, 3].

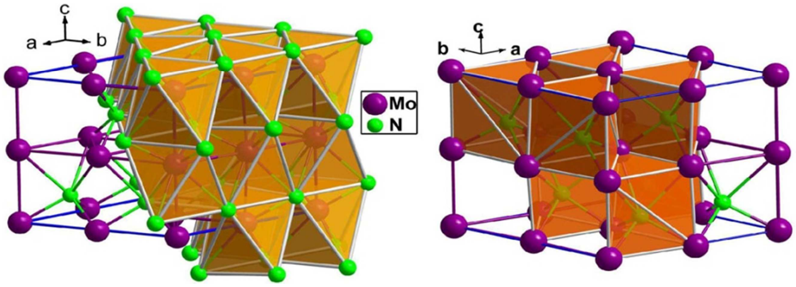

Fig. S4. (**Left**) Polyhedral views of crystal structures of hexagonal δ–MoN; the Mo atoms are octahedrally coordinated with N atoms (*i.e.,* [MoN6]. (**Right**) Nitrogen atoms are in a distorted trigonal–prismatical coordination with Mo atoms (*i.e.,* [NMo_6]).

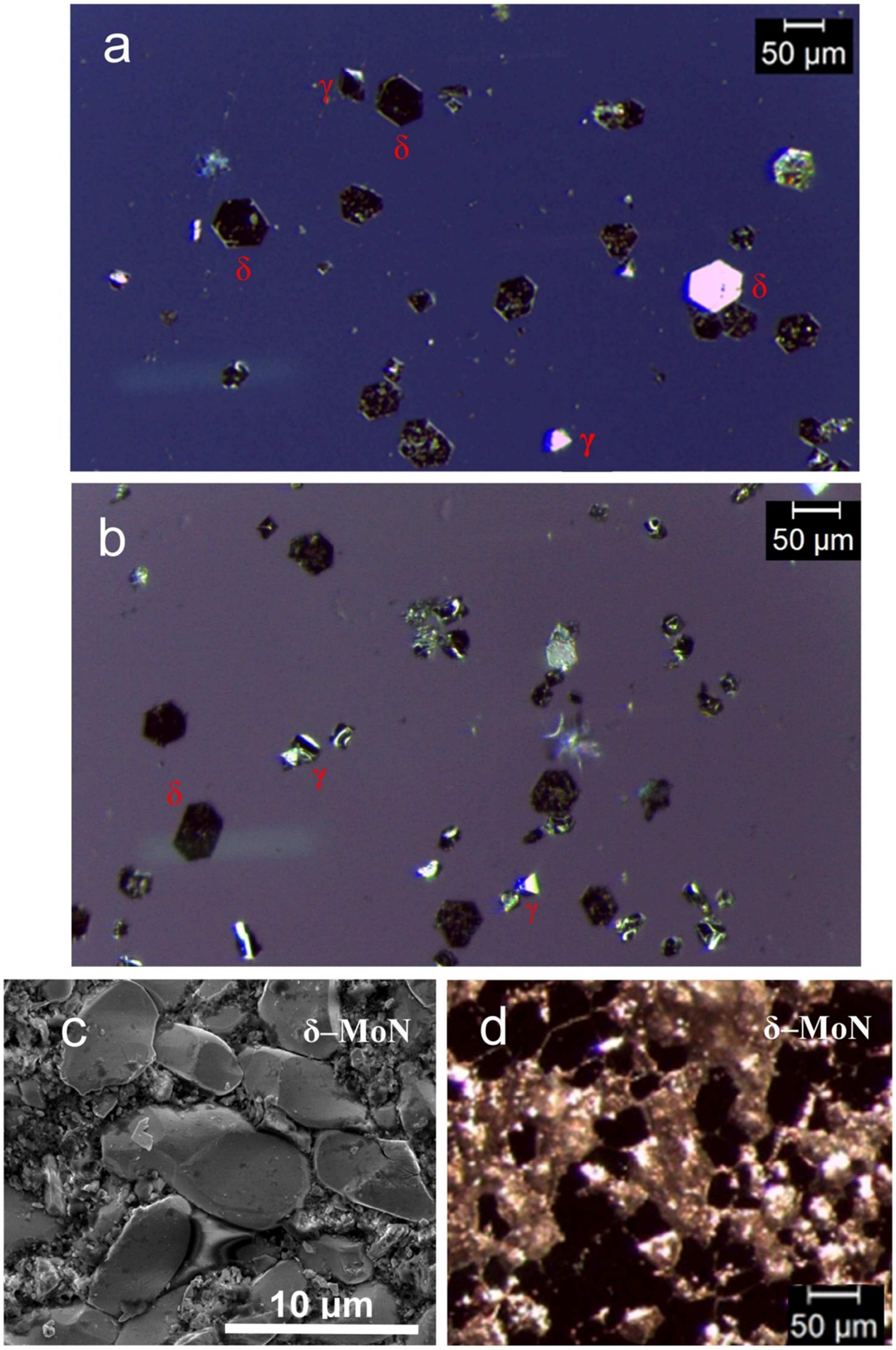

Fig. S5. (**a**)–(**b**) Optical images of γ– and δ–MoN single crystals grown at 3 GPa by program–controlled heating for 3 hours. (**c**) δ–MoN crystals obtained by re–sintering of phase–pure δ–MoN powders at 5 GPa and 1400 °C for 30 min. (**d**) An optical

image of δ–MoN bulk sample sintered at 8 GPa and 1800 °C for 45 min. The black areas in (d) are polished surfaces for δ–MoN crystals. XRD measurements indicate that the sintered crystalline bulks in (c) and (d) are phase–pure δ–MoN.

2. Compression measurement

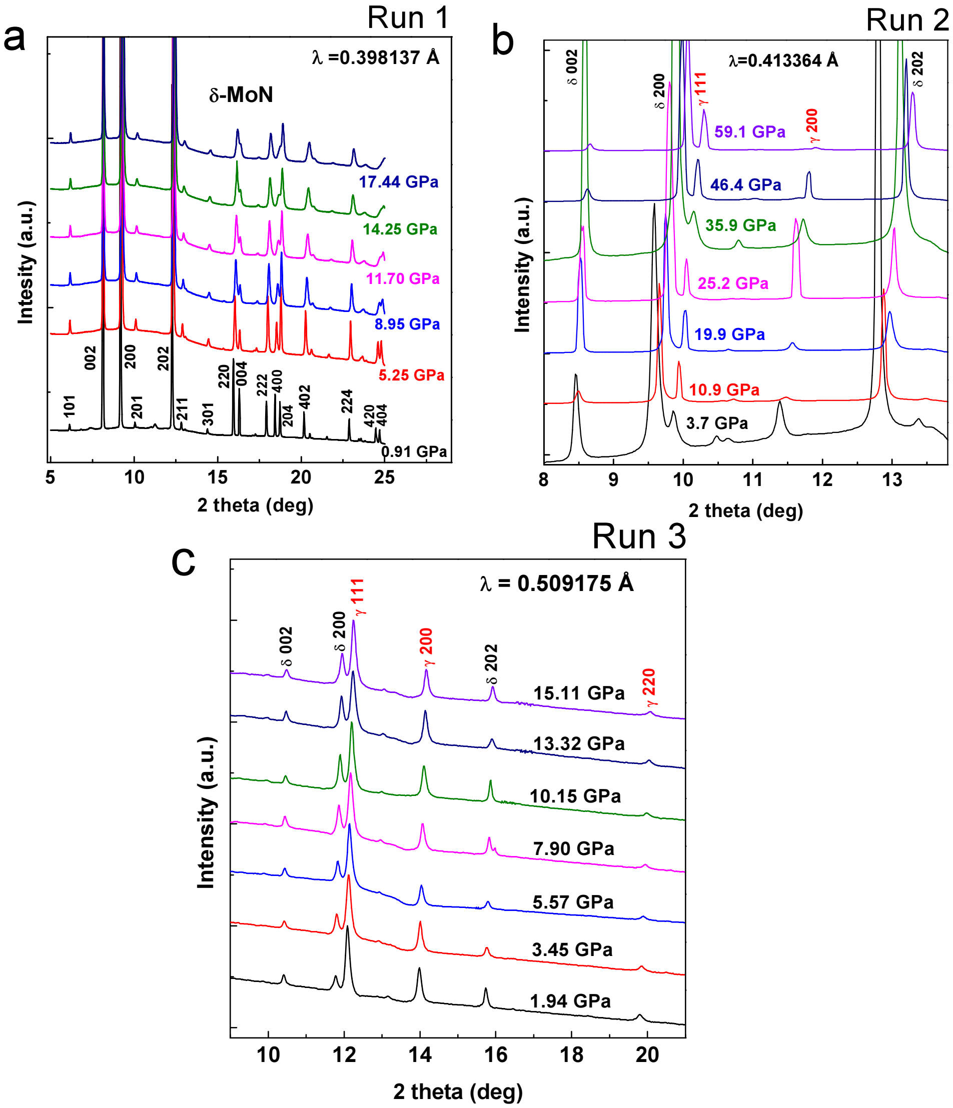

Fig. S6. Selected high–P x–ray diffraction patterns for δ– and γ–MoN from three independent experimental runs. (**a**) for phase–pure δ–MoN. (**b**) and (**c**) for coexisting δ and γ phases. The cubic phase in (b) is substoichiometric γ–$MoN_{0.86}$.

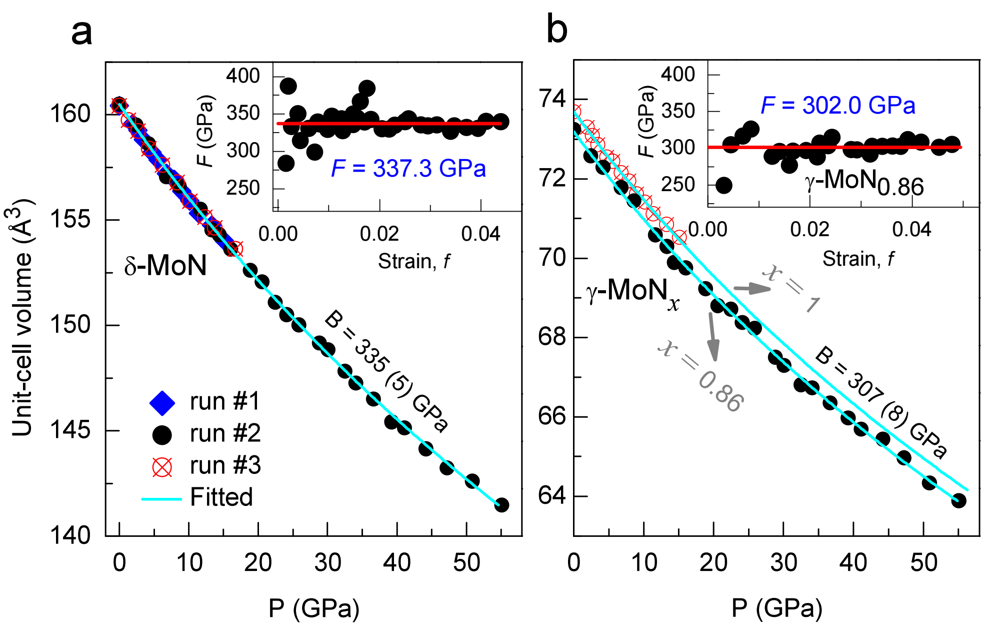

Fig. S7. P–V data for δ–MoN (a) and γ–MoN$_x$ (x = 0.86 and 1) (**b**). The insets show the relationships between the normalized pressure (F) and Eulerian strain (f), given by,

$$F = P \cdot [3f \cdot (1 + 2f)^{2.5}]^{-1} \qquad (S1)$$

$$f = \frac{1}{2}\left[\left(\frac{V}{V_0}\right)^{-\frac{2}{3}} - 1\right] \qquad (S2)$$

where P, V, and V_0 are experimental pressure, unit–cell volume at a pressure P, and unit–cell volume at ambient conditions, respectively. The data in the F–f plots indicate that the pressure derivative of bulk modulus, $B' = \partial B/\partial P$, is ~4 for both δ and γ phases, and the fitted constants, F, are 337.3 and 302.0 GPa, respectively. The obtained P–V data were also fitted to the 2nd order Birch–Murnaghan equation of state,

$$P(V) = 3B_0 \cdot f \cdot (1 + 2f)^{\frac{5}{2}} \qquad (S3).$$

The thus–derived bulk moduli, B_0, for both phases are 335 (5) and 307 (8) GPa, respectively, which agree well with the obtained F values. It is worthwhile to mention

that the determined B_0 for sub–stoichiometric γ–$MoN_{0.86}$ is very close to that of γ–MoN.

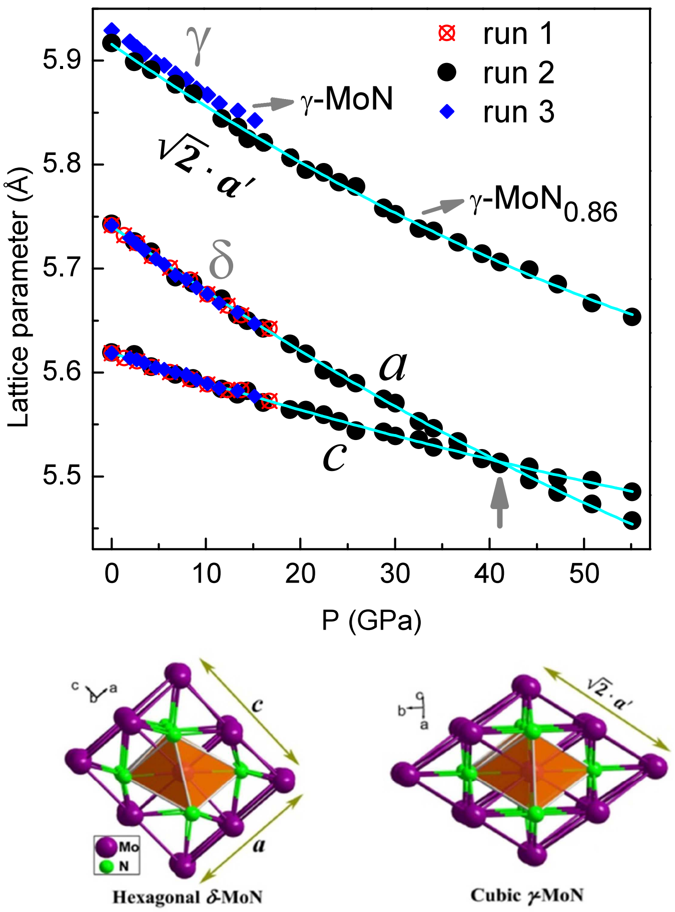

Fig. S8. (Upper panel) Axial compressibilities as a function of pressure for δ–MoN, γ–$MoN_{0.86}$, and γ–MoN. The normalized lattice parameter, x/x_0 (x = a and c), fitted to a truncated Fourier series expansion of the form,

$$\frac{x}{x_0} \approx 1 + \xi \cdot \left(\frac{P}{P_0}\right) + \eta \cdot \left(\frac{P}{P_0}\right)^2 \quad (S4).$$

For simplicity, we define P_0 = 1 GPa to derive dimensionaless constants of ξ and η. Such derived constants are listed in Table S1. (**Bottom panel**) Polyhedral views of crystal structures of δ– and γ–MoN.

Table S1. Derived constants ξ and η for δ–MoN, γ–$MoN_{0.86}$, and γ–MoN. The reported values for WC are also listed for comparison.[4]

	δ-MoN		γ-MoN_x (x = 0.86 and 1)		WC[4]	
	ξ ($\times10^{-4}$)	η ($\times10^{-6}$)	ξ ($\times10^{-4}$)	η ($\times10^{-6}$)	ξ ($\times10^{-4}$)	η ($\times10^{-6}$)
$\frac{a}{a_0}$	-11.20	3.95	-10.5	4.60	-8.30	1.60
$\frac{c}{c_0}$	-5.21	1.64	-	-	-6.10	1.60

The obtained η is two orders of magnitude smaller than ξ, indicating that the pressure deviation of lattice strain (x/x_0) can mainly been determined by ξ. As shown in Table S1, δ–MoN has a high axial incompressibility along c–axis (–5.21), which is even stiffer than that in WC (–6.10), one of the most incompressible substances.

3. Low–T superconductivity measurement

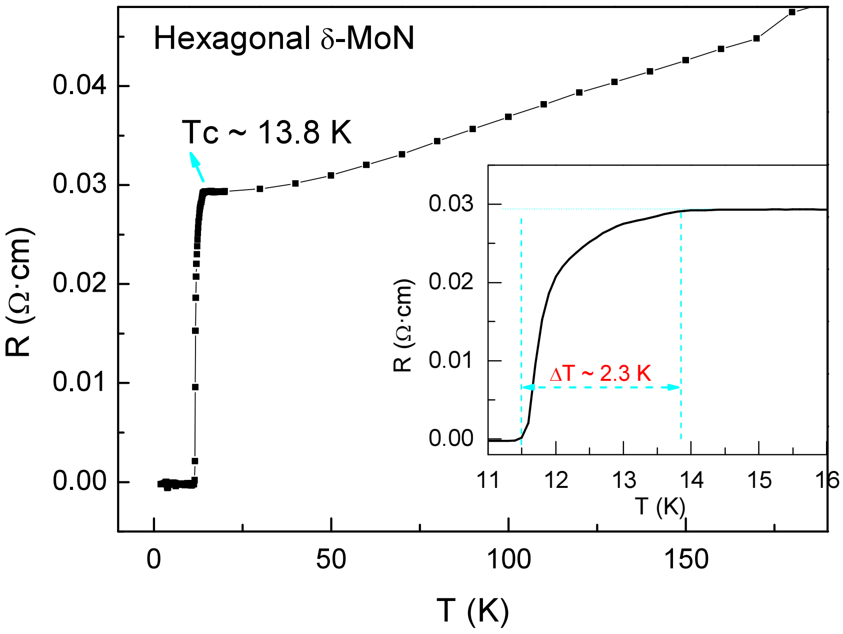

Fig. S9. Low–T four–probe resistivity measurement for well–sintered, polycrystalline

hexagonal δ–MoN (see Fig. 2d in text). Inset is an enlarged portion near the superconducting transition temperature. The transition width, ΔT, is determined to be ~2.3 K.

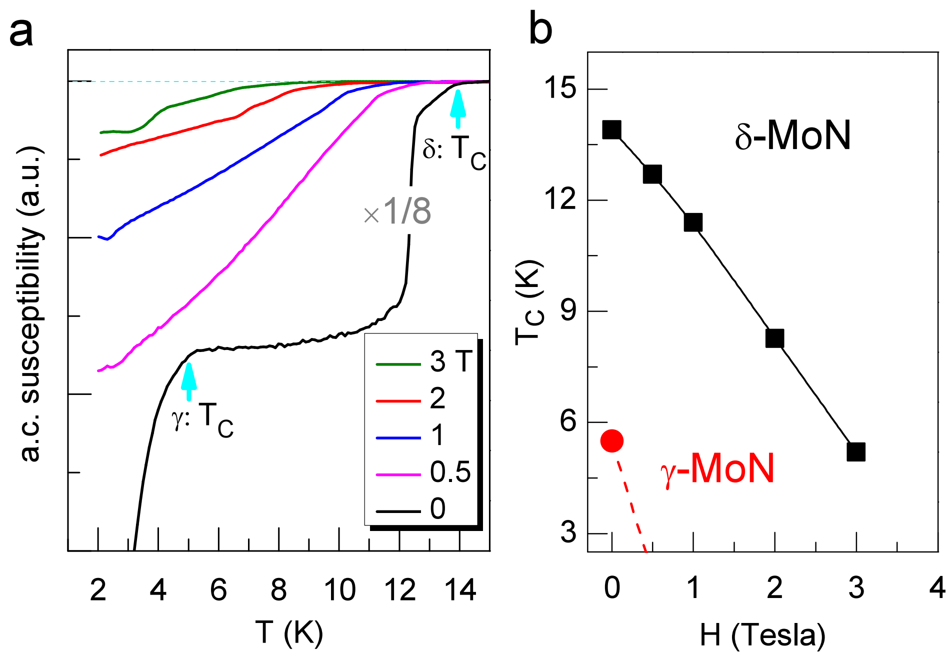

Fig. S10. (**a**) Temperature dependence of magnetic susceptibility for δ– and γ–MoN collected under different magnetic fields of H = 0, 0.5, 1, 2, and 3 Tesla. (**b**) T_c as a function of applied field for the two phases of MoN.

References:

1. Toby, B.H. EXPGUI, a graphical user interface for GSAS. *J. Appl. Cryst.* **34**, 210-213 (2001).
2. Machon, D. *et al.* High pressure-high temperature studies and reactivity of γ-Mo2N and δ-MoN. *Phys. Status Solidi A* **203**, 831-836 (2006).
3. Bull, C.L. *et al.* Crystal structure and high-pressure properties of γ-Mo2N determined by neutron powder diffraction and X-ray diffraction. *J. Solid State Chem.* **179**, 1762-1767 (2006).
4. Lin, Z. *et al.* Nanocrystalline tungsten carbide: As incompressible as diamond. *Appl. Phys. Lett.* **95**, 211906 (2009).
